# Frontoparietal anodal tDCS reduces ketamine-induced oscillopathies

**DOI:** 10.1515/tnsci-2020-0157

**Published:** 2021-06-28

**Authors:** Caroline Lahogue, Didier Pinault

**Affiliations:** Université de Strasbourg, Strasbourg, France; INSERM U1114, Neuropsychologie Cognitive et Physiopathologie de la Schizophrénie, Strasbourg, France; Fédération de Médecine Translationnelle de Strasbourg (FMTS), Centre de Recherche en Biomédecine de Strasbourg (CRBS), Faculté de médecine, Strasbourg, France

**Keywords:** delta oscillations, gamma oscillations, NMDA receptors, non-REM sleep, psychosis transition, quantitative EEG, pentobarbital, spindles, thalamus, transcranial electrical stimulation

## Abstract

During the prodromal phase of schizophrenia with its complex and insidious clinical picture, electroencephalographic recordings detect widespread oscillation disturbances (or oscillopathies) during the wake–sleep cycle. Neural oscillations are electrobiomarkers of the connectivity state within systems. A single-systemic administration of ketamine, a non-competitive NMDA glutamate receptor antagonist, transiently reproduces the oscillopathies with a clinical picture reminiscent of the psychosis prodrome. This acute pharmacological model may help the research and development of innovative treatments against psychotic transition. Transcranial electrical stimulation is recognized as an appropriate non-invasive therapeutic modality since it can increase cognitive performance and modulate neural oscillations with little or no side effects. Therefore, our objective was to set up, in the sedated adult rat, a stimulation method that is able to normalize ketamine-induced increase in gamma-frequency (30–80 Hz) oscillations and decrease in sigma-frequency (10–17 Hz) oscillations. Unilateral and bipolar frontoparietal (FP), transcranial anodal stimulation by direct current (<+1 mA) was applied in ketamine-treated rats. A concomitant bilateral electroencephalographic recording of the parietal cortex measured the stimulation effects on its spontaneously occurring oscillations. A 5 min FP anodal tDCS immediately and quickly reduced, significantly with an intensity-effect relationship, the ketamine-induced gamma hyperactivity, and sigma hypoactivity at least in the bilateral parietal cortex. A duration effect was also recorded. The tDCS also tended to diminish the ketamine-induced delta hypoactivity. These preliminary neurophysiological findings are promising for developing a therapeutic proof-of-concept against neuropsychiatric disorders.

## 1 Introduction

Effective treatments against chronic schizophrenia without side effects are still missing [1,2]. Its development takes years with the occurrence of prodromal symptoms associated with attention-related sensorimotor and cognitive deficits [3], dysfunctional brain networks [4,5], and widespread oscillation disturbances [6,7]. These prodrome-related oscillopathies include an excessive amplification of broadband gamma-frequency (30–80 Hz) oscillations [8] and a reduction in the density of sleep slow-wave oscillations and spindles [9,10,11,12,13,14]. Neural oscillations, naturally implicated in attentional and integrative processes, are biomarkers of the connectivity state within systems. Proton magnetic resonance spectroscopy reveals, during the prodrome, a decrease in glutamate and glutamine levels [15,16], which are correlated with gray matter volume in the frontoparietal (FP) system. These findings support the glutamate hypothesis of schizophrenia [2,17].

Aberrant amplification of broadband gamma oscillations can be reproduced in cortical and subcortical structures in healthy humans and rodents after a single-systemic administration, at a psychotomimetic dose, of the *N*-methyl-d-aspartate glutamate receptor antagonist ketamine [18,19,20,21]. The state and function of cortical and subcortical networks are altered, including in the FP corticothalamic system, which plays an essential role in attentional and integrative processes. Furthermore, the ketamine-elicited gamma hyperactivity decreases the ability of cortico-thalamo-cortical networks to integrate incoming information [22,23]. In pentobarbital-sedated rats (sleep-like state), ketamine transiently reduces the power of slow-wave oscillations and spindles by switching the firing pattern of both thalamic relay and reticular neurons from burst mode to the single-action potential mode [24]. Furthermore, clozapine, one of the most effective antipsychotic medications currently available, especially in treatment-resistant patients with schizophrenia [25,26], prevents the ketamine effects on thalamocortical slow-wave oscillations and spindles [24]. Therefore, the ketamine-induced oscillopathies represent translational electrical biomarkers for cerebral network disorders with prognostic and therapeutic potential, a hope for the research and development of innovative treatments against psychotic transition.

Transcranial direct current stimulation (tDCS) is recognized as an appropriate non-invasive therapeutic modality since it can increase cognitive performance and modulate neural oscillations with little or no side effects [27,28,29,30]. This stimulation makes it possible to modulate the physiological or pathological cortical and subcortical activities in humans [31,32,33] and rodents [34]. In healthy volunteers at rest, functional magnetic resonance imaging reveals that bipolar FP or frontotemporal anodal tDCS modulates corticostriatal and corticothalamic connectivity [32,33]. In addition, at the level of the primary motor cortex, tDCS can modulate the different nodes of the cortico-thalamo-cortical circuit. In addition, FP tDCS makes it possible to reduce positive [35] and negative [36] symptoms in patients with schizophrenia who are resistant to antipsychotics. Additionally, in patients with schizophrenia, anodal tDCS can reduce the gamma event-related synchronization [37]. Also, the use of tDCS has a great potential in the treatment of cognitive symptomatology in early psychosis [38]. Nevertheless, the tDCS-induced immediate and downstream effects are transient and the parameters of tDCS remain to be further investigated to better understand their impact on network and cellular activities.

Our objective was to set up a preclinical, experimental FP tDCS design allowing the refinement of the parameters under well-controlled conditions while recording the ongoing parietal EEG oscillations. We used subcutaneous tDCS (electrodes directly positioned on the skull), a technical design that is more efficient in modulating the firing of cortical and subcortical neurons as it frees from the shunting effects of the skin and head musculature surrounding the skull [34,39]. The bipolar format (nearby stimulating electrodes) was privileged as it results in more localized current flow than monopolar DCS (two remote stimulating (brain) and reference (e.g., body) electrodes), which stimulates a larger volume of brain tissue [40]. We also applied unilateral FP tDCS in an attempt to appraise the local and distant effects of the bipolar tDCS. For this, we used the pentobarbital-sedated rat, a model of slow-wave sleep with spindle-like activities and bouts of gamma oscillations [24], which made it possible to quantitatively apprehend the stimulation effects on the ketamine-induced oscillopathies. The present, conceptually, and data-driven pilot study shows that unilateral FP anodal tDCS was substantially efficient in reducing these ketamine-induced effects.

## 2 Methods and materials

### 2.1 Animals and drugs

Fourteen Wistar adult male rats (285–370 g) were used. Ketamine (Imalgene^®^ 1000), Fentanyl (Fentadon^®^), lidocaine (Lurocaine^®^), and pentobarbital (Euthasol^®^) were provided from Centravet (Nancy, France).


**Ethical approval:** The research related to animals’ use has been complied under the approval of the Ministère de l’Enseignement Supérieur, de la Recherche et de l’innovation.

### 2.2 Surgery under deep narco-analgesia

Narcosis was initiated with an intraperitoneal injection of pentobarbital (60 mg/kg). An additional dose (10–15 mg/kg) was administered as soon as there was a nociceptive reflex. Analgesia was achieved with a subcutaneous injection of fentanyl (7–10 μg/kg) every 30 min. The depth of the surgical narco-analgesia was continuously monitored using an electrocardiogram, watching the rhythm and breathing, and assessing the nociceptive withdrawal reflex. The rectal temperature was maintained at 36.5°C (peroperative and protective hypothermia) using a thermoregulated pad. The trachea was cannulated and connected to a ventilator (50% air–50% O2, 60 breaths/min). Under local anesthesia (lidocaine), an incision of the skin on the skull was done, and the periosteum was removed to set the skullcap bared and to perform the stereotaxic positioning of the stimulating and recording electrodes on the FP skull. The deep narco-analgesia lasted about 2.5 h, the time needed to complete all the surgical procedures.

### 2.3 Analgesic pentobarbital-induced sedation

At the end of the surgery, the body temperature was set to and maintained at 37.5°C. The analgesic pentobarbital-induced sedation (light narco-analgesia) was initiated about 2 h after the induction of the surgical narco-analgesia and was maintained by a continuous intravenous infusion of the following regimen (average quantity given per kg and per hour): Pentobarbital (4.2 ± 0.1 mg), fentanyl (2.4 ± 0.2 μg), and glucose (48.7 ± 1.2 mg). In order to help maintain the ventilation stable and to block muscle tone and tremors, a neuromuscular blocking agent was used (d-tubocurarine chloride: 0.64 ± 0.04 mg/kg/h). The cortical EEG and heart rate were under continuous monitoring to adjust, when necessary, the infusion rate to maintain the sedation. The EEG recordings began 2 h after the beginning of the infusion of the sedative regimen. During the recording session and every 2 h, drops of the local anesthetic lidocaine were applied to the surgical wounds.

### 2.4 Unilateral FP tDCS combined with bilateral cortical EEG

**Figure 1:**
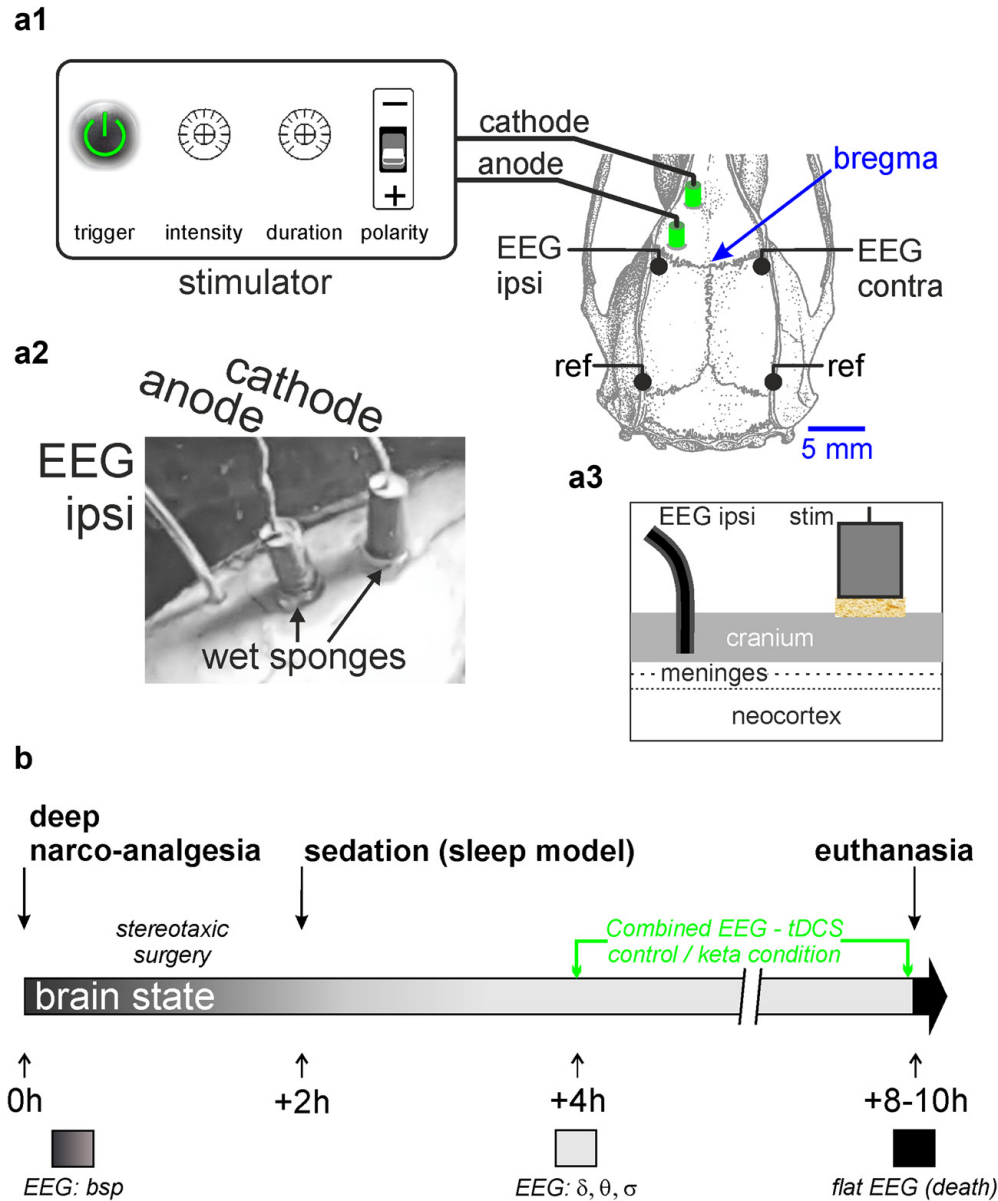
Experimental design. (a1) Dorsal view of the rat skull showing the location of the EEG recording electrodes positioned on the parietal somatosensory cortex, and the stimulation electrodes, the cathode at the frontal level and the anode at the parietal level. The references of the EEG electrodes are positioned on the occipital ridges. (a2) Photography of the bipolar stimulation electrodes and of the ipsilateral EEG electrode (EEG ipsi) relative to the location of the stimulation electrodes. (a3) Diagram illustrating the location, non-invasive from the point of view of the brain, of the EEG electrodes, the surface of each electrode in contact with the inner plate of the skull, and the stimulation electrodes, each lying on a sponge soaked in saline solution and pressed on the surface of the skull. (b) Timeline illustrating the key events during the experimental procedure. The color code of the brain state is dark gray for deep narco-analgesia, light gray for sedation (light narco-analgesia), and dark for death. bsp, Burst Suppression Pattern. During the sedation, the EEG is characterized prominently by delta-, theta- and sigma-frequency oscillations (*δ*, *θ*, and *σ*, respectively).

For the unilateral, bipolar tDCS, we used pellet Ag/AgCl electrodes (Warner Instruments), 1.5 mm in diameter and 3 mm in height. The electrodes were positioned on the left side of the skull, the cathode above the frontal area (relative to Bregma: anterior: 5 mm; lateral: 1 mm), and the anode above the parietal area (anterior: 3 mm; lateral 3 mm) (Figures 1a1 and a2). In an attempt to reduce the inhomogeneities of electrical conductivity of the skin–skull interface [41] and its substantial shunting effect (up to 75%) [34], the skull was slightly drilled at the electrode placement areas (about 2 mm in diameter), a strategy that secured the electrode position. The electrodes were positioned on wet sponges (NaCl, 0.9%) 2 mm in diameter and 1.0 mm in thickness (Figures 1a2 and a3). Drops of saline solution were regularly applied to the sponges to keep them moist, a strategy to minimize the electrical shunting effects, which was expected to maintain as stable as possible the current flow during the application of the stimulating current. The electric current was supplied using a Master-8 stimulator (A.M.P.I.) equipped with an isolation unit to deliver a constant current. At the end of the acute experiment, the animals were killed during a lethal administration of Euthasol^®^.

For the bilateral cortical EEG, four recording Teflon-sheathed silver wires (diameter: 200 μm) were implanted in the skull. Four slight drill holes were done up to the internal plate of the skull where the recording section of the wires was in contact. The two, right and left, active electrodes were placed in the parietal skull over the primary somatosensory cortex (from Bregma: 2.3 mm posterior; 5 mm lateral), and the references (ground mode) were positioned on the occipital ridges. The EEG signals (0.1–800 Hz) were acquired using an ultralow-noise differential amplifier (AI 402, ×50; Molecular Devices). All signals were sampled at 10 kHz 16-bit (Digidata 1440A with pCLAMP10 Software, Molecular Devices).

### 2.5 Repeated measures in one animal

As long as the pentobarbital-induced sedation is stable (4 to 6 h) and knowing that, under the present experimental conditions, the ketamine effect (peaking at 15–20 min) significantly lasts less than 90 min [23,24], two to three protocols (saline alone, ketamine alone, tDCS alone, and/or ketamine combined with tDCS) could be performed in one animal (Figure 1b and Table S1). Each animal was its own control, meaning that, for a given protocol, every rat was exposed to the control condition (at least 20 min before ketamine administration) and then to the condition of interest (ketamine or/and ketamine + tDCS).

### 2.6 Data analysis

Analysis software packages Clampfit v10 (Molecular Devices) and SciWorks v10 (Datawave Technologies) were used. Spectral analysis of baseline EEG oscillations was performed with the fast Fourier transformation (FFT, 0.5 Hz resolution). The power of EEG activities was analyzed in three frequency bands: delta (1–4 Hz), sigma (10–17 Hz, spindles), and gamma (30–80 Hz). For each band, the total power was the sum of all FFT values. Power measures were averaged into 2 min blocks (60 values ± SEM) given as a percentage of change from the averaged values under the control condition (about 100%). Tested parameters and number of rats used for each condition are presented in Table S1. Statistical analyses were performed using the software R v3.6.1 (R Core Team, Vienna Austria, 2019). Comparison between vehicle condition (tDCS 0 mA) and tDCS condition was done using parametric tests: paired student’s *t*-test and one-way analysis of variance with a Tukey’s posthoc test HSD (“honestly significant difference,” significance level *p* < 0.05). The Wilcoxon test was used where data were not normally distributed. Each animal was its own control.

## 3 Results

Under the sleep-like, ketamine-free condition, the EEG recordings displayed spontaneous and predominant oscillations in the delta-frequency band (1–4 Hz or slow waves) accompanied by oscillations in the sigma band (10–17 Hz or “spindle-like” activities) [24,42]. These oscillatory activities had characteristics qualitatively similar to slow-wave sleep with spindles recorded in free-behaving rats in stage II sleep. The slow-wave sleep-type oscillations were sometimes interspersed with smaller and faster oscillations including, among others, broadband gamma- and higher-frequency oscillations.

The parametrization of the FP tDCS began according to an empirical and pragmatic approach (Table S1). This strategy allowed us to quickly move toward another stimulation protocol (a new combination of parameters) that we predicted to be effective based on the ongoing datasets, our knowledge, and expertise. The experimental conditions were stable and reliable enough over time, giving the possibility to apply up to three protocols, thereby adjusting the stimulation parameters during every experiment and from one to the next experiment, a refinement strategy that gives potentially useful results with a reasonably low number of animals. More specifically, each experiment was followed by a spectral analysis of strategic EEG segments and a debriefing to decide the stimulation parameters to apply to the next rat. Four rats were used to optimize the efficiency of the wet sponge to minimize the potential electrical shunting effect of the electrode–skull interface (Figures 1a1–a3) to get a stable and reliable stimulation effect. This was assessed on the basis of the ipsi- and contralateral potential responses evoked following a 1 ms pulse of electrical microstimulation with an intensity varying from −0.03 to −0.60 mA (two rats, Figure S2). After a short latency (about 1 ms), an evoked potential was recorded and, on the contralateral parietal cortex, followed by a prominent sigma-frequency oscillation lasting about 1 s (Figure S2). The evoked contralateral oscillation, highlighted after averaging, was visible from −0.30 mA. The spectral analysis revealed a significant increase (Wilcoxon test, *p*-value = 0.00873) in the power of the sigma oscillations, which corresponded to an evoked spindle-like activity (Figure S2).

Our objective is to set up a FP tDCS capable of reducing or normalizing the effects of ketamine on spontaneously occurring neuronal oscillations, all the results presented in the following were obtained in the ketamine condition, that is, after a single subcutaneous administration of ketamine at a subanesthetic and psychotomimetic dose (2.5 mg/kg) [19]. It is worth reminding that, in the sedated rat, ketamine fleetingly decreases the power of delta oscillations and spindles (sigma-frequency oscillations) and increases that of broadband gamma- and higher-frequency oscillations with a peak effect 15–20 min after its systemic administration [24]. A partial or total recovery is usually observed 60–80 min later. In the present study, an overview of the overall effect of ketamine is presented in Figure S3. Among the 14 rats, four were excluded from the data analyses because the sponge interface pads were not wet enough and/or properly positioned, which impacted the quality of the tDCS current flow ascertained by the presence of many artifacts.

### 3.1 FP anodal tDCS

**Figure 2:**
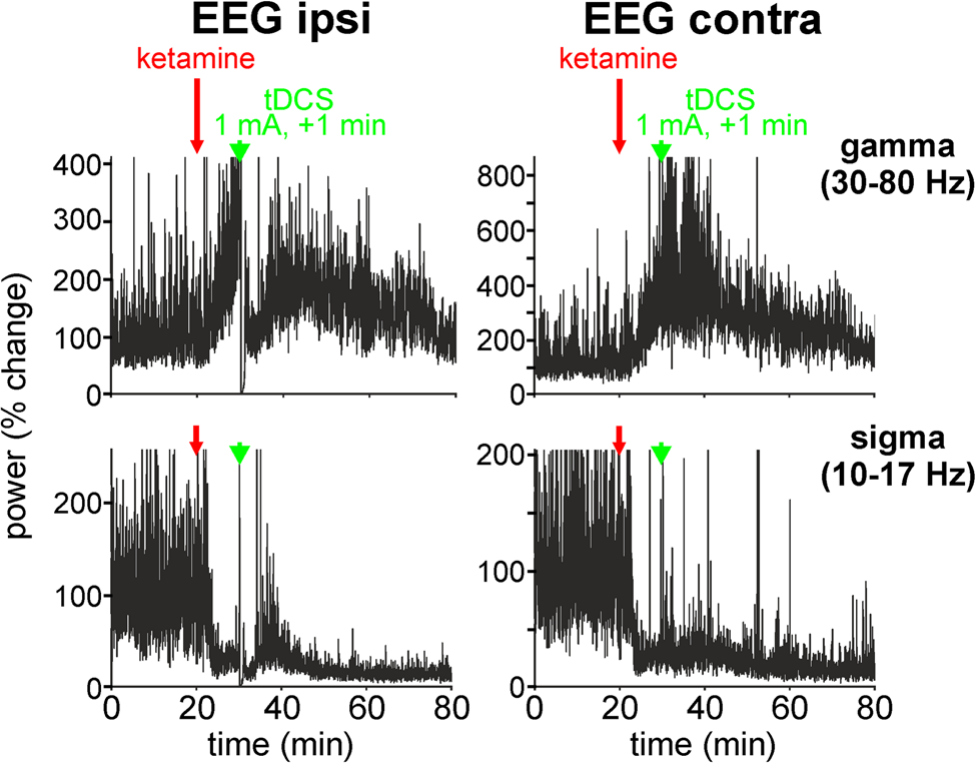
Anodal tDCS of +1 mA for 1 min transiently modulates the power of the ketamine-induced bilateral, gamma hyperactivity, and sigma hypoactivity. The stimulation is applied 10 min after (at time 20 min) the systemic administration of ketamine (2.5 mg/kg), that is, when the ketamine-induced sigma hypoactivity and gamma hyperactivity are well installed. The values (one per 2 s) presented are in % change in power.

We started to seek an intensity capable of modulating immediately, quickly, and substantially the pattern of EEG oscillations. For this, a 1 min FP tDCS with increasing intensity was applied either from 0 to +1 mA or from 0 to −1 mA (with 0.2 mA increments) on one rat, and from +0.5 to +1 mA (0.5 mA increment) on a second rat. In each rat, the first stimulation was applied 10 min after the administration of ketamine, that is, about 5 min before its peak effect (at 15–20 min postinjection) on cortical gamma oscillations. The minimum time interval in between two successive tDCS was 8 min. A tDCS of ±1 mA was, immediately, able to fully or transiently saturate the amplifier of the ipsilateral EEG. The EEG took on the appearance of an isoelectric trace, whereas the contralateral EEG was slightly or not affected (Figure S4). The spectral analysis of the cortical EEG activities reveals (Figure 2) the following: (i) a transient normalization in the ketamine-induced gamma hyperactivity for about 5 min, an effect that was more remarkable in the ipsilateral than the contralateral EEG and (ii) a decrease in the power of the concomitant spindle-like activity lasting approximately 2 min followed by a transient increase for about 5 min, an effect also more remarkable in the ipsilateral than the contralateral EEG. These results were reproducible in another rat, leading us to further investigate the FP anodal tDCS with an intensity inferior to +1 mA and by increasing the duration (>1 min).

### 3.2 Duration effect

**Figure 3:**
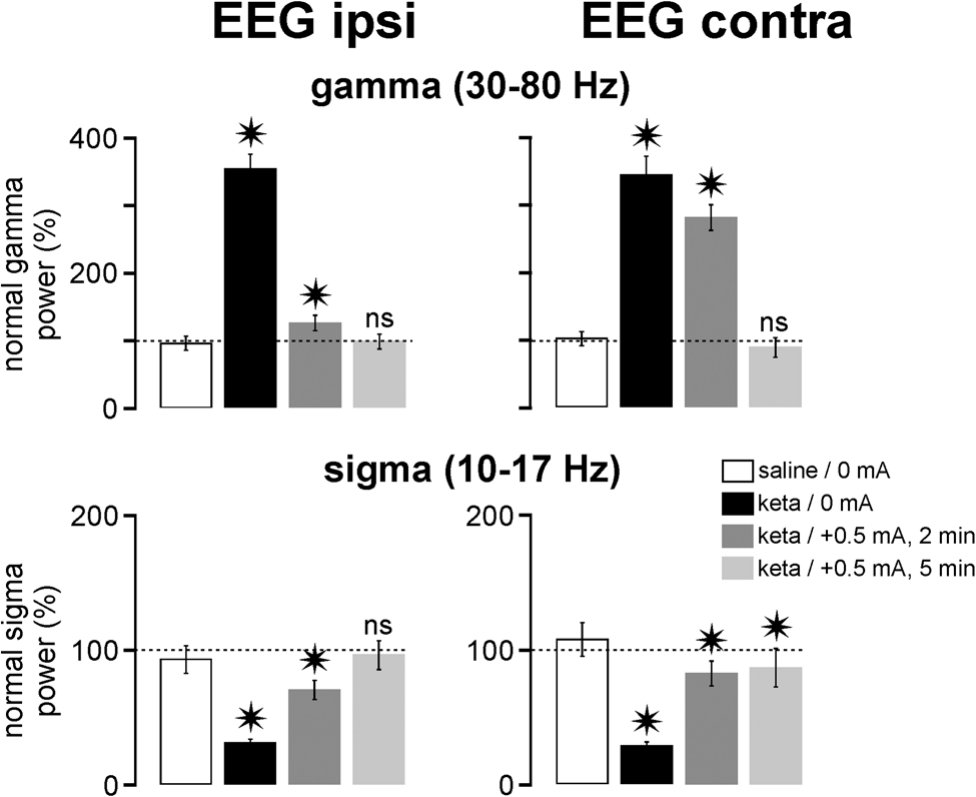
Duration effect of tDCS on ketamine-induced gamma hyperactivity and sigma hypoactivity. The spectral analysis (FFT or fast Fourier transform) is performed for 2 min 5 min after the end of the tDCS. Data are normalized. The ketamine effect (keta/tDCS 0 mA) and each of the keta/tDCS conditions +0.5 mA (2 or 5 min) are compared to the “saline/0 mA” control (one rat per condition, 60 values/rat, each rat being its own control). Student’s *t*-test (ns, non-significant; *p < 0.05). *p*-values (comparisons relative to saline/0 mA): Up left histogram: *p* < 0.00001 (keta/0 mA); < 0.00001(keta/+0.5 mA, 2 min); 0.41635 (keta/+0.5 mA, 5 min); Up right: *p* < 0.00001 (keta/0 mA); < 0.00001 (keta/+0.5 mA, 2 min); 0.13115 (keta/+0.5 mA, 5 min); Down left: *p* < 0.00001 (keta/0 mA); 0.00023 (keta/+0.5 mA, 2 min); 0.33902 (keta/+0.5 mA, 5 min). Down right: *p* < 0.00001 (keta/0 mA); = 0.00094 (keta/+0.5 mA, 2 min); 0.01594 (keta/+0.5 mA, 5 min).

The duration effect was assessed with a FP tDCS of +0.5 mA in two rats, one rat per duration (2 and 5 min). The stimulation was applied 10 min after ketamine administration. The 2 min spectral analysis was carried out 5 min after the end of the tDCS, that is, at the peak effect time of the ketamine-elicited gamma hyperactivity. The results are presented in Figure 3. The effectiveness of the tDCS in reducing the ketamine-induced gamma hyperactivity increased when increasing the duration. More specifically, a full normalization was recorded in both the ipsi- and the contralateral EEGs after an anodal tDCS (+0.5 mA) lasting 5 min.

Regarding the spindle-like activities, the tDCS +0.5 mA, with a duration of 2 or 5 min, tended to normalize the ketamine-induced reduction in spindle power in the bilateral EEG (Figure 3). The effectiveness of the stimulation in the ipsilateral EEG was higher with a duration of 5 min than with 2 min, whereas both durations had almost the same effect in the contralateral cortex. A complete normalization (non-significant relative to the saline condition) was recorded in the ipsilateral EEG with a tDCS of 5 min. So, these observations led us to set the duration of the tDCS at 5 min.

### 3.3 When to apply bipolar tDCS?

**Figure 4:**
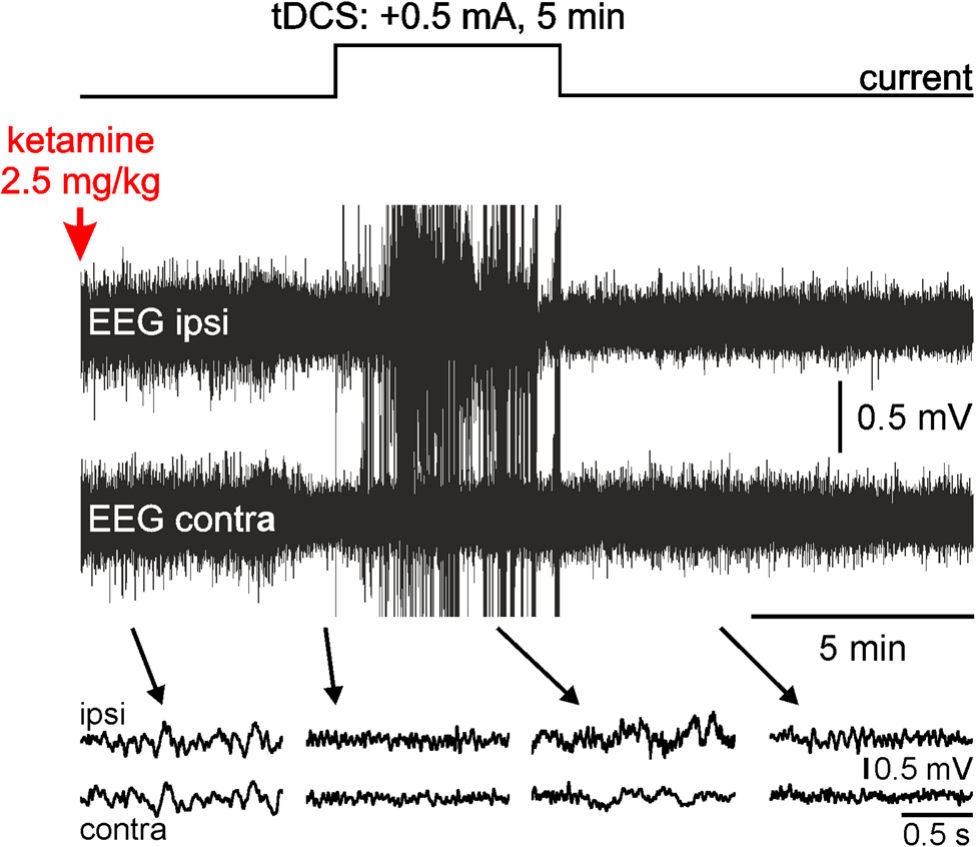
Anodal (+0.5 mA) tDCS for 5 min modulates the EEG oscillation patterns. In pentobarbital-sedated rats, the bilateral EEG exhibits a sleep-like pattern with slow oscillations, mainly slow waves in the delta frequency band (1–4 Hz), and brief (0.5–1.5 s) oscillations in the sigma band (10–17 Hz, spindle-like activities). Ketamine begins to transform EEG slow-waves into faster and less ample EEG waves 4–5 min after its systemic administration. The tDCS is applied 5 min after ketamine administration. During the stimulation, the EEG ipsilateral to the stimulation electrodes is strongly altered with numerous artefacts.

**Figure 5:**
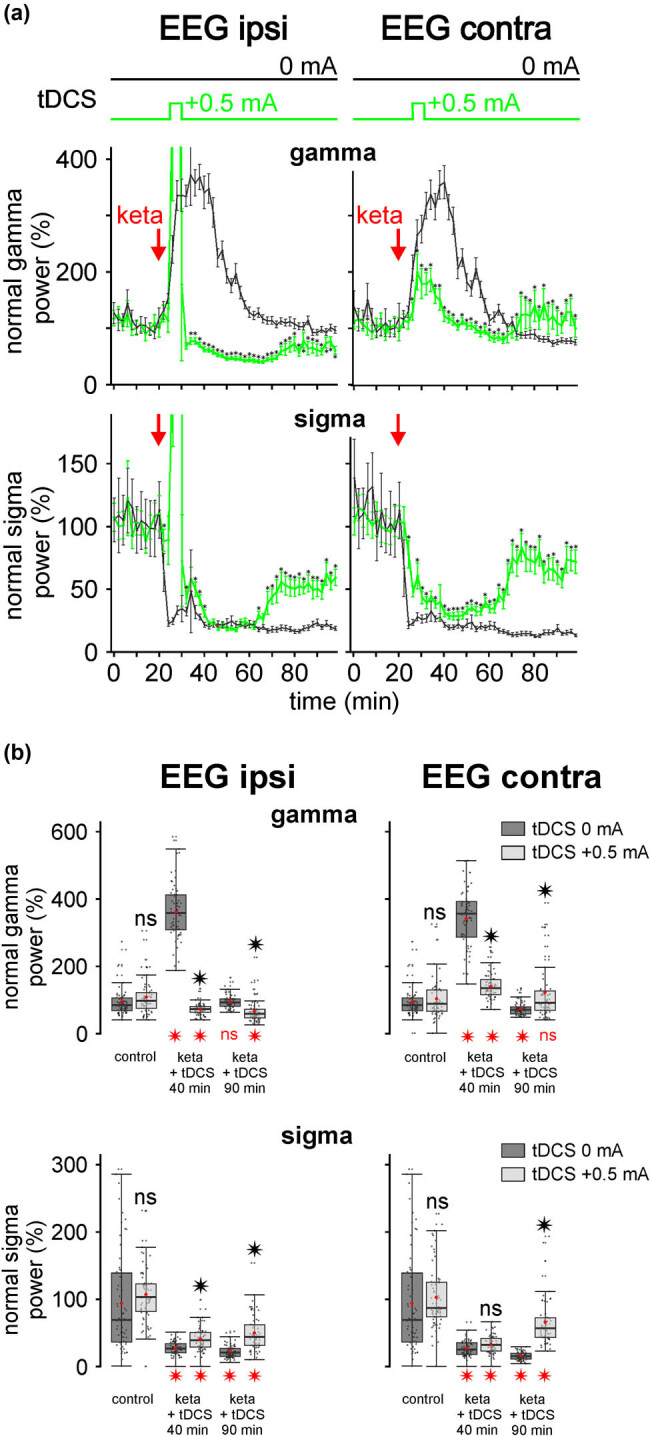
(a) The bipolar anodal tDCS (+0.5 mA, 5 min) bilaterally reduces the ketamine peak effect. In black (“sham control”, one rat): effect of ketamine (ketamine-tDCS 0 mA) on the normalized power of the gamma (top) and sigma (bottom) oscillations. In green (another rat): tDCS was applied 5 min after the subcutaneous injection of ketamine (2.5 mg/kg) and for 5 min (25 to 30 min). The values of the power of the oscillations are represented with a resolution of 2 min (each point is an average of 60 values, ±standard error of the average). The effect of the stimulation is compared to the ketamine-DCS (0 mA) control group. Student’s *t*-test (*p < 0.05). (b) Boxplots from the data presented in the graphs of the Figure 5a. One rat per condition. Dots represent the distribution of 60 values for each condition. Red point: mean; thick black line: median; bottom and top of the box, first and third quartile, respectively; error bars: ±standard deviation. Two student’s *t*-tests (ns, non-significant, *p < 0.05) were done. The one in black (above): comparison of ketamine-tDCS 0 mA with ketamine-tDCS +0.5 mA. Ipsi gamma (from left to right): *p* = 0.13086; <0.00001; *p* < 0.00001. Contra gamma (from left to right): *p* = 0.37492; <0.00001; <0.00001. Ipsi sigma: *p* = 0.56024; <0.00001; <0.00001. Contra gamma: *p* = 0.82378; = 0.06530; <0.00001. The red one (bottom): comparison of each box dataset to the saline condition (control, ∼100%). Ipsi gamma (from left to right): *p* < 0.00001; =0.00039; =0.98885; =0.00001. Contra gamma: *p* < 0.00001; <0.00001; <0.00001; =0.14098. Ipsi sigma: *p* <0.00001; <0.00001; =0.02509; <0.00001. Contra sigma: *p* < 0.00001; <0.00001; <0.00001; <0.00001.

So far, we applied our stimulation 10 min after the systemic administration of ketamine, that is, when its peak effect on cortical gamma oscillations started to emerge. In a previous study, it was demonstrated that clozapine, one of the most effective antipsychotic medications currently available, prevented the ketamine effects [24]. So, with the idea to prevent the ketamine-induced oscillopathies, we wanted to test, in another rat, whether an earlier tDCS was capable in deleting the ketamine peak effect. For this, we applied 5 min tDCS +0.5 mA 5 min after ketamine administration (Figure 4). At the time of ketamine injection, the EEG was relatively synchronous, containing delta oscillations and spindles. The bipolar anodal tDCS was applied when (5 min postinjection) the ketamine effects started to be visible (Figure 4). During the stimulation, atypical slow waves occurred, accompanied with many artifacts (amplifier saturations), especially in the ipsilateral EEG. The atypical waves and artifacts disappeared immediately after the end of the stimulation. The tDCS significantly suppressed the ketamine-induced peak of gamma hyperactivity (at 35–40 min) completely in the ipsilateral EEG (*p* < 0.00001) and partially in the contralateral EEG (*p* < 0.00001; Figure 5a and b). In this rat, the stimulation was ipsilaterally so powerful that it significantly reduced the gamma power below the normal value (100%) recorded under the saline condition. The same anodal tDCS +0.5 mA tardily and significantly (*p* < 0.001 at 85–90 min) reduced the ketamine-induced spindle hypoactivity in the bilateral cortical EEG without reaching a normalization.

### 3.4 The minimum effective intensity

**Figure 6:**
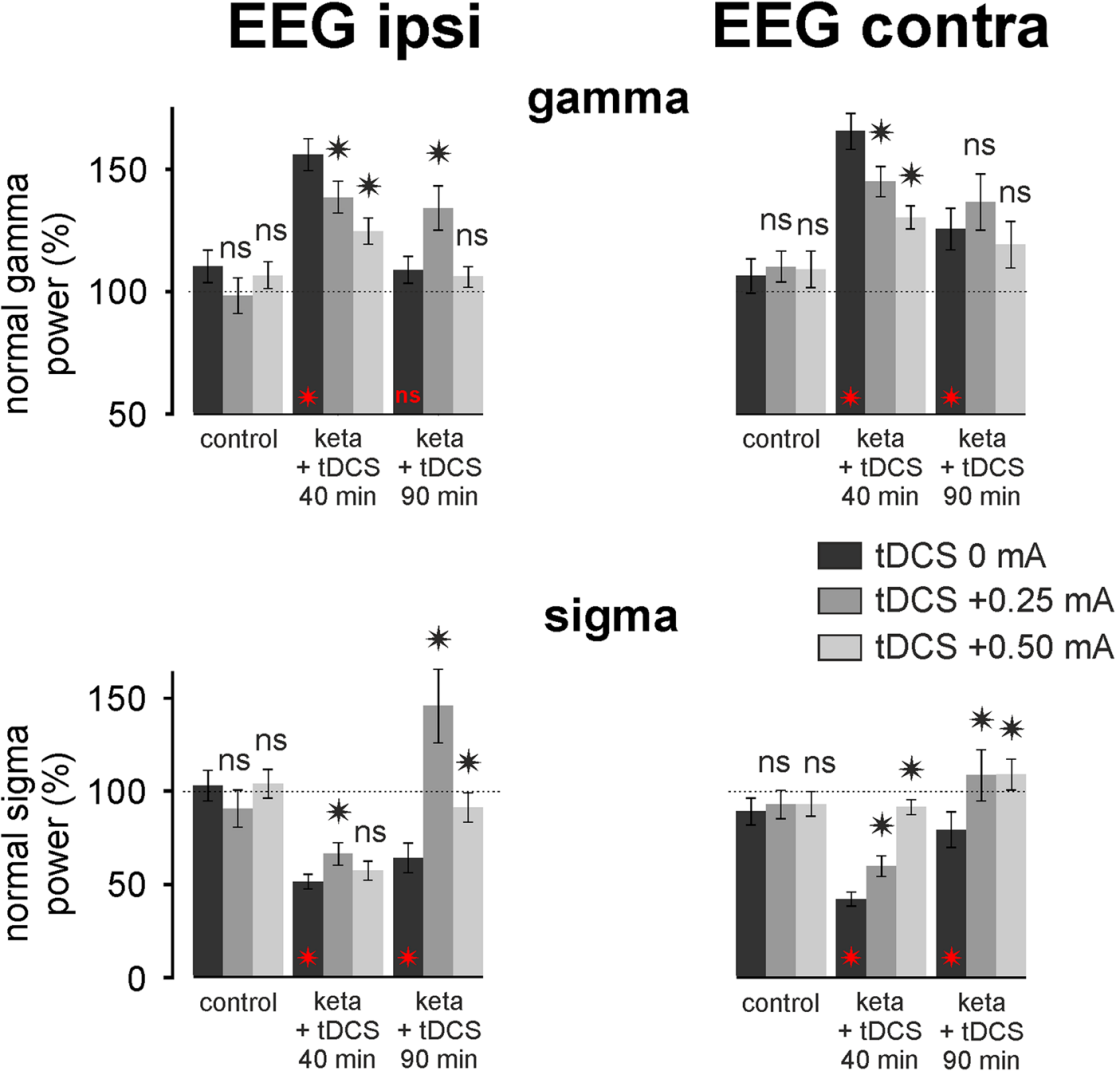
Intensity effect of tDCS on the ketamine-induced oscillopathies. The tDCS +0.25 or +0.50 mA was applied 8 min after the systemic administration of ketamine with three rats per condition (ketamine + tDCS 0, +0.25 or +0.50 mA). Each column represents the percentage of the normalized power of the gamma or sigma oscillations (average of three rats × 60 values over 2 min ± SEM) for each of the conditions: control (18–20 min), ketamine + tDCS 40 min (38–40 min) and at 90 min (88–90 min). *t*-test comparison, relative to the ketamine-tDCS 0 mA condition, of the ketamine-tDCS effect +0.25 or +0.50 mA (ns, not significant; * *p* < 0.05). Ipsi gamma (from left to right): *p* = 0.06759; =0.68321; =0.00017; <0.00001; =0.00015; =0.68744. Contra gamma (from left to right): *p* = 0.42845; =0.60073; =0.00004; <0.00001; =0.12329; =0.31399. Ipsi sigma: *p* = 0.71709; =0.85512; =0.00111; =0.36876; <0.00001; =0.00001. Contra sigma: *p* = 0.06643; =0.85109; <0.00001; <0.00001; =0.00069; <0.00001. In red: *t*-test comparison, relative to the control condition (saline, 100%), of the ketamine effect (tDCS 0 mA) at 40 and 90 min after the ketamine administration. Ipsi gamma (from left to right): *p* < 0.00001; =0.57550. Contra gamma (from left to right): *p* < 0.00001; =0.00065. Ipsi sigma: *p* < 0.00001; <0.00001. Contra sigma: *p* < 0.00001; =0.02719.

**Figure 7:**
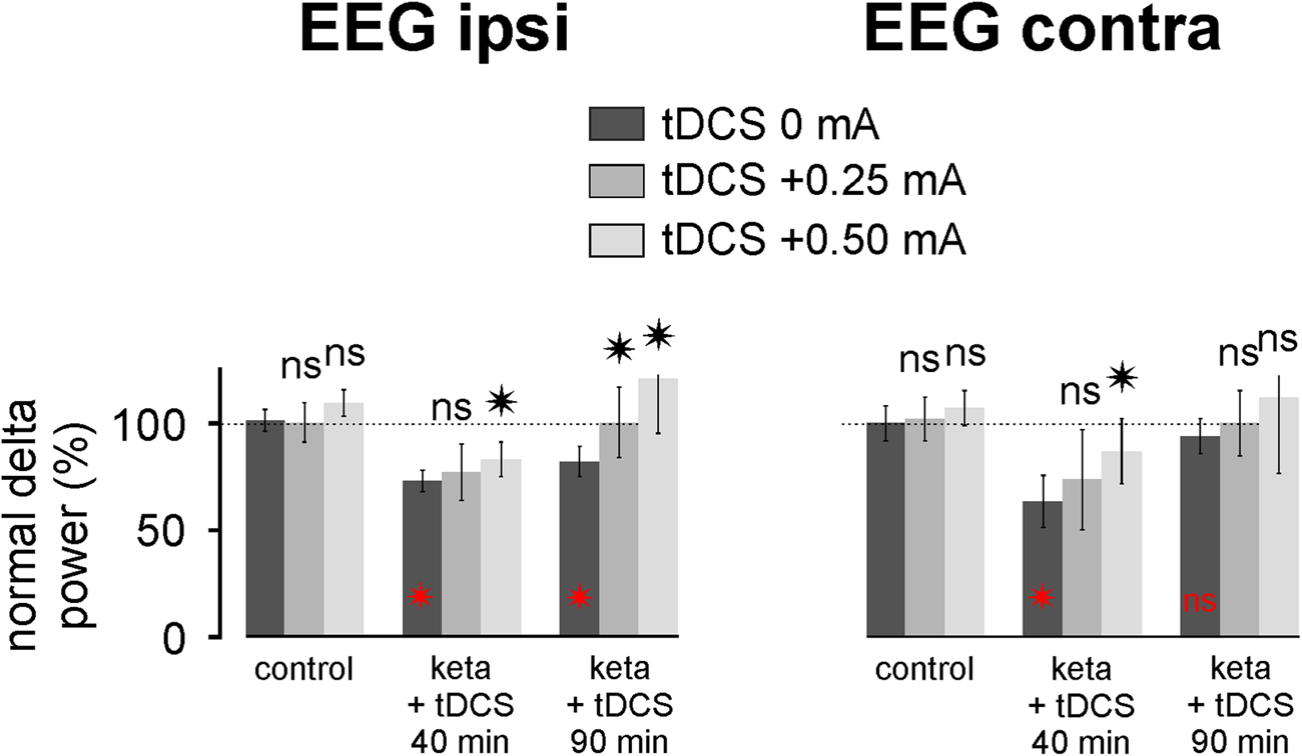
The transcranial frontoparietal DCS tends to reduce the ketamine effects on delta oscillations. The tDCS +0.25 or +0.50 mA was applied 8 min after the systemic administration of ketamine with three rats per condition (ketamine + tDCS 0, +0.25 or +0.50 mA). Each column represents the percentage of the normalized power of the delta oscillations (average of three rats × 60 values over 2 min ± SEM) for each of the conditions: control (18–20 min), ketamine + tDCS 40 min (38–40 min) and at 90 min (88–90 min). *t*-test comparison, relative to the ketamine-tDCS 0 mA condition, of the ketamine-tDCS effect +0.25 or +0.50 mA (ns, not significant; * *p* < 0.05). *p*-values EEG ipsi: 0.0136 (keta +40 min, tDCS +0.50 mA); 0.0047 (keta +90 min, tDCS +0.25 mA); 0.00023 (keta +90 min, tDCS +0.50 mA). *p*-values EEG contra: 0.00881 (keta +40 min, tDCS +0.50 mA). In red: *t*-test comparison, relative to the control condition (saline, 100%), of the ketamine effect (tDCS 0 mA) at 40 and 90 min after the ketamine administration. *p*-values EEG ipsi: 0.00001 (keta +40 min); 0.00001 (keta +90 min). *p*-values EEG contra: 0.00001 (keta +40 min).

Here, it is shown that exogenous currents had an instantaneous influence on ongoing brain activities, which is in agreement with previous comprehensive studies [43,44]. So, it was important to assess the minimum effective intensity in reducing or normalizing the ketamine-induced oscillopathies. For this, we compared the effects of the FP anodal tDCS +0.25 and +0.50 mA for 5 min with three rats per condition (Figure 6). The tDCS was applied 8 min after ketamine administration. A one-factor analysis of variance revealed an intensity effect in the bilateral EEG for both the ketamine-induced gamma hyperactivity (ipsilateral EEG: *F* = 23.48, *p* < 0.001; contralateral EEG: *F* = 17.82, *p* < 0.001) and the concomitant spindle hypoactivity (ipsilateral EEG: *F* = 42.94, *p* < 0.001; contralateral EEG: *F* = 36.27, *p* < 0.001). The tDCS +0.5 mA was more effective at 40 min than the tDCS +0.25 mA in bilaterally reducing the ketamine-induced gamma hyperactivity and in contralaterally reducing the concomitant spindle hypoactivity. A significant difference (*p* < 0.05) between the +0.25 mA and +0.5 mA groups was observed. Because the tDCS was efficient in reducing ketamine-induced spindle hypoactivity, we expected that the tDCS could similarly reduce the ketamine-induced delta hypoactivity. Figure 7 shows that the tDCS tended to diminish the delta hypoactivity. The efficacy was significant at 40 min with +0.5 mA both ipsi- and contralaterally and at 90 min for both +0.25 and +0.50 mA in the ipsilateral EEG.

## 4 Discussion

The present preclinical pilot investigation provides promising technical and neurophysiological essentials for developing a non-invasive therapeutic proof-of-concept against the transition to a psychotic state. A 5 min FP anodal tDCS with an intensity of less than +1 mA can, immediately and quickly, reduce, with intensity and duration effects, the ketamine-induced gamma hyperactivity and spindle hypoactivity. It tended to also reduce the associated delta hypoactivity. Technical, neurophysiological, neurochemical, and structural issues deserve discussions for the implementation of in-depth studies aiming at optimizing tDCS methods.

### 4.1 Experimental conditions

Our experiments were carried out in rats under narcosis induced by pentobarbital, which elicits a slow-wave sleep with spindle-like activities by increasing the GABAergic neurotransmission [24,45]. The pentobarbital preparation was stable and adjustable at will via EEG-driven infusion rate. The major requirement of the present study was to have a stationary stage II non-REM sleep, during which we could perform repeated measures using a minimal number of animals. These experimental conditions, relatively stable over time (at least up to 8 h), are “ideal” for determining and adjusting the multiple and various parameters of the electrical stimulation. Indeed, they made it possible to control, in a durable and relatively reliable way from one to another animal, a given parameter while recording the brain activities before, during, and after the stimulation. They also help us, throughout the experiments, to perform repeated measures and thereby to optimize the stimulation parameters. Such a goal is almost impossible to swiftly achieve in the free-behaving animal, that is, to obtain a high success rate and reliable results in a reasonable time. Such a risky strategy would lead to a sacrifice of a large number of animals and would require years of research with questionable financial and human costs. The present datasets remain preliminary as it was impossible to test, in a reasonable time, all the possible combinations (extraordinarily large number) of the stimulation parameters. The present pilot study may play a pivotal role in planning comprehensive studies at a reasonable cost.

Both the skin and the skull form a complex mechanical and bioelectric interface with the variability of skull conductivity and thickness, which can lead to inter-individual variability [41]. So, in our pilot investigation, we attempted to set up a simpler and more reliable preparation in an attempt to optimize, as precisely as possible, the multiple stimulation parameters (duration, intensity, polarity). We securely placed our stimulation electrodes on saline-soaked sponges directly on the skull, which was slightly drilled at the electrode placement areas. Under these experimental conditions, a tDCS with an intensity of less than 1 mA was expected not to damage the structure and anatomical properties of intracortical neurons, first because of the shunting effects of the wet sponges, and second because the stimulation influence on the bilateral cortical EEG oscillations was reversible. Independently, an elegant study demonstrated that tDCS intensities up to 0.8 mA do not generate injury discharges in cortical neurons [34].

### 4.2 Technical considerations

In the present series of experiments, the unilateral FP tDCS was very useful to evaluate its local (ipsilateral EEG) and distant (contralateral EEG) effects. The latter effect likely included a multi-synaptic influence mediated in great part by the callosal pathway. Regarding the recordings of the rat illustrated in Figure 5, it is clear that tDCS +0.5 mA was immediately more effective in correcting the ketamine-induced gamma hyperactivity in the ipsilateral than the contralateral cortex. In contrast, the efficacy of tDCS in reducing the ketamine-induced spindle hypoactivity was more evident in the contralateral than the ipsilateral cortical EEG. As a single 1 ms stimulation evoked more spindle-like activities in the contralateral than in the ipsilateral cortex (see Figure S2), it would make sense to conclude that the efficacy of the tDCS on the contralateral spindle-like activities is secondary to the primary ipsilateral effects. Nevertheless, based on our previous data recorded under the same experimental conditions [24], it is questionable whether the late (about 40 min after ketamine administration) cortical activities were due either to a “true” stimulation effect (ketamine combined with tDCS +0.5 mA effects) or to a spontaneous partial recovery (ketamine alone (tDCS 0 mA)). The present group data do not exclude an efficacy of the FP anodal tDCS paralleled with partial recovery. So, further investigation is necessary to clarify this point.

Furthermore, our results show, at the group level, an intensity effect (0, +0.25, and +0.50 mA, three rats per condition) of a 5 min FP anodal tDCS, indicating that the stimulation could partially or completely normalize the ketamine-induced oscillopathies, regarding at least the sigma- and broadband gamma-frequency oscillations. In humans, since the duration of the stimulation can be up to a few tens of minutes [35,46,47], it would be logical to seek a minimum effective intensity perhaps by increasing either the duration of the stimulation or the contact surface of the stimulating electrodes. Here, it was shown that the unilateral tDCS induced a functional imbalance between the ipsi- and contralateral cortices. Therefore, an interhemispheric or a bilateral stimulation is expected to be a better alternative, perhaps even with a lower intensity, which would correct this imbalance by influencing equally the hemodynamic and neurophysiological activities on both sides.

The present study does not, nevertheless, offer a standardized tDCS method. Here, we assessed the local and distant effects of a unilateral, FP anodal tDCS on the acute ketamine-induced oscillopathies. With the bipolar format, further investigation is required to probe other options. For instance, as the frontal and parietal cortical areas are reciprocally connected, it would be logical to probe also a parieto-frontal anodal tDCS, the cathode at the parietal level, and the anode at the frontal level. Also, knowing that anodal and cathodal tDCS can have similar effects on synaptic plasticity [48], it would also be wise to investigate the effects of FP and PF cathodal tDCS.

One major limitation is the dependence of tDCS on brain state [49,50]. So, the tested parameters set under pentobarbital-induced sedation highlight the importance of considering the brain state when it comes to adjust the stimulation parameters in any, experimental or clinical, studies.

### 4.3 Functional and mechanistic aspects

Under our experimental conditions, the unilateral FP anodal tDCS exerted local and remote influences on ongoing cortically and thalamically generated activities. This is in agreement with the available relevant literature. Indeed, in the rat, it was comprehensively demonstrated that, in contrast to a transcutaneous stimulation, a subcutaneous tDCS significantly modulates the firing and membrane potential of cortical neurons [34]. It was also demonstrated that DCS can change the polarization of axon terminals, thereby affecting the action potential dynamics and modulating the synaptic efficacy [51]. In humans, the DCS-induced electrical field spreads rapidly throughout the brain and modulates, locally and remotely, short- and long-range mono/multisynaptic cortical and cortical-subcortical systems [31,32,33,52,53]. Furthermore, it was demonstrated that anodal tDCS of the frontal cortex widely increases cerebral blood flow in many cortical and subcortical structures [52] and can enhance sleepiness and sleep-related EEG oscillations [54]. However, the cell-to-network mechanisms underlying the immediate and downstream effects of regional tDCS remain to be elucidated. They may include immediate and in-cascade, short- and medium-term network, synaptic, cellular, and molecular processes, including multiple plasticity processes [30,48,55,56,57]. A computational study predicts that focal stimulation can trigger new functional large-scale neural connections [58]. The effects of tDCS also depend on the state of the brain and neural systems [52,59,60,61,62,63,64]. The thalamus might be implicated in the tDCS effects as high-frequency electrical stimulation of the FP thalamocortical pathway exerts effects that are opposite to those of the NMDA receptor antagonist ketamine, especially simultaneously on both the synaptic plasticity and the gamma power [22]; experimental findings support the notion that electrical stimulation of the thalamus has pro-sensory/cognitive properties [65,66].

**Figure 8:**
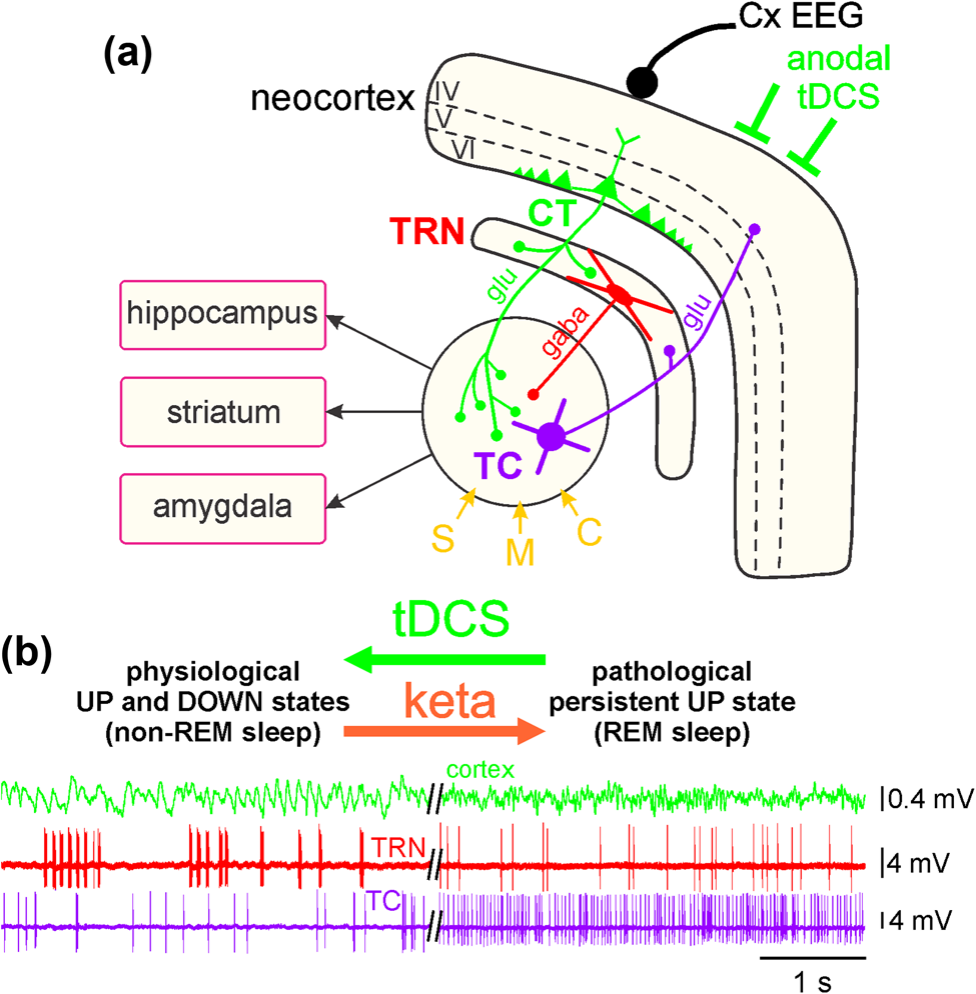
Theoretical prediction of the cell-to-network effects of a frontoparietal anodal tDCS in the corticothalamic system. (a) Simplified drawing of the hodology of the 3-neuron CT-TRN-TC circuit. The layer 6 corticothalamic (CT) and thalamocortical (TC) neurons are glutamatergic while the thalamic reticular nucleus (TRN) neuron is GABAergic. Both TC and CT axons innervate the TRN. This system receives sensory (S), motor (M), and cognitive/associative (C) inputs. It is important to specify that the layer VI CT neurons outnumber by a factor of about 10 the TC neurons. (b, left) Physiological UP and DOWN states: during the non-REM sleep, the TC system displays principally a synchronized state, characterized by the occurrence of delta oscillations and spindles; the TRN cell exhibits mainly rhythmic (at the delta-, theta- and sigma-frequency bands) high-frequency bursts of action potentials. The synchronized state includes two sub-states, UP and DOWN, which are usually associated with active and quiescent cellular firings, respectively. (b, right) Pathological persistent UP state: This ketamine-induced persistent UP state is assumed to be an abnormal REM sleep. After a single systemic administration of a subanesthetizing low-dose of ketamine, the TC system displays a more desynchronized state (peak effect at about +15–20 min) characterized by the prominent occurrence of lower voltage and faster activities (>16 Hz), which include beta-, gamma- and higher-frequency oscillations. Under the ketamine condition, both the TC and the TRN neurons exhibit a persistent irregular and tonic firing containing more single APs than high-frequency bursts of APs. The bipolar anodal tDCS is expected to reduce, even to normalize, the ketamine-induced oscillopathies. Adapted from Mahdavi et al., Schizophr Res, 2020.

Furthermore, from the EEG recordings of the parietal cortex, we can make testable predictions regarding the underlying cellular and synaptic activities of the thalamic neurons. Indeed, broadband gamma oscillations and spindles result from functional synaptic interactions between GABAergic and glutamatergic neurons. More specifically, gamma oscillations implicate such synaptic interactions in both the cortex and the thalamus [67,68]; sleep-related spindles are generated principally in the thalamus with synaptic interactions between the thalamic relay and reticular neurons [24,69]. So, when the EEG is synchronized, it predominantly displays delta oscillations and spindles, and the corresponding thalamic relay and reticular neurons mainly fire rhythmic high-frequency bursts of action potentials [69]. When the EEG is desynchronized, it predominantly exhibits faster and lower amplitude oscillations, including broadband gamma-frequency oscillations, and the corresponding thalamic relay and reticular neurons principally fire single action potentials in the tonic irregular mode. In the sedated rat, ketamine transiently reduces the power of delta oscillations and spindles and increases in power broadband gamma- and higher-frequency oscillations by switching the firing of both relay and reticular neurons from the burst mode to the single-action potential mode [24]. Therefore, we predict that, at least in the FP corticothalamic system of the ketamine-treated sedated rat, the FP anodal tDCS increases the power of delta oscillations and spindles and decreases broadband gamma- and higher-frequency oscillations by switching the firing of the thalamic glutamatergic and GABAergic neurons from the single-action potential mode to the burst mode. This prediction can be tested using a cell-to-network electrophysiological exploration (Figure 8), which may help to understand some aspects of the mechanisms underlying the tDCS-induced change in the state of the concerned neural systems.

Previous studies support the notion that tDCS can modulate the membrane activity of neurons [48], in particular through glutamatergic NMDA receptors and GABAergic receptors [70,71]. The anodal stimulation would depolarize the membrane potential and increase neuronal excitability, in particular by reducing intracortical inhibition and increasing paired-pulse excitability [47]. On the other hand, cathodal stimulation would hyperpolarize the membrane potential and decrease neuronal excitability through its inhibitory action on glutamatergic neurons [70,71,72].

The blockade of NMDA receptors by ketamine prevents the expression of long-term potentiation in rodents [22]. The tDCS can modulate the synaptic plasticity with effects that depend on the spatial and temporal properties of synapses [48,73]. We can hypothesize that since tDCS can reduce the abnormal oscillations induced by ketamine, it will also be able to durably reduce the ketamine-induced decrease in long-term potentiation (LTP), in turn modulating LTP or long-term depression through the glutamatergic and GABAergic receptors. Further investigation is necessary to decipher the mechanisms underlying the impact of FP anodal tDCS on ketamine-induced oscillopathies.

### 4.4 Outlook

The present, conceptually and data-driven pilot study brings key findings that may help, through comprehensive studies, the standardization of tDCS methods in animal models of psychosis transition and, perhaps, in individuals having a high-risk state for psychosis. The present findings may also help to understand the neural effects of antipsychotic drugs such as clozapine.

At the preclinical level, after the primary phase of scientific and technological innovations, it would be rational to investigate the unilateral or bilateral tDCS in the ongoing brain activities of free-behaving rodents under physiological and pathological (e.g., under ketamine influence) conditions. The atypical antipsychotic clozapine is efficient in reducing, at least in the rodent, the ketamine- or MK-801-induced oscillopathies [24,74,75,76]. Therefore, the acute ketamine model seems appropriate to assess the potential preventive and/or curative effects of tDCS. In the second phase of research and development, it would be logical to explore the tDCS in more realistic models of psychotic transition, for instance of genetic-neurodevelopmental types. This would certainly help to appreciate whether tDCS could correct or normalize not only the brain oscillopathies but also the associated psychosis-relevant behavior and cognitive impairment. Indeed and interestingly, tDCS during adolescence, before psychosis-relevant behavioral abnormalities, prevents the development of positive symptoms in the rodent maternal immune stimulation model of schizophrenia [77].

At the clinical level, one may ask questions regarding the potential clinical application of the tDCS in individuals having an insidious clinically at-risk mental state (ARMS) to develop, usually within 2 years, a transition to psychosis while our ketamine model acutely and transiently induces a brain state simulating in part that of patients transitioning to psychosis. So, which would be the best parameters, when, and how many times tDCS could be applied in ARMS individuals to delay or prevent a psychotic transition? This is a challenging question as to the acute and possible chronic effects of a single or repetitive tDCS in such individuals remains to be known, especially as a transition to psychosis can occur within 2 years in one-third of ARMS individuals [78]. Also, is an oscillation-driven tDCS sufficiently efficient in preventing the occurrence of a first psychotic state? Should tDCS be tested on baseline or functional (e.g., task or sensory-related) oscillations, which are also disturbed in the early phase of schizophrenia [79]? Like behavior-to-cognitive variables, functional oscillations would be appropriate as both cognitive processes and brain oscillations are altered in ARMS individuals [8,80,81]. However, the oscillopathies recorded in ARMS patients are subtler than those recorded during a ketamine-induced psychosis-relevant brain state in both humans and rodents [82]. The behavior-to-cognitive variables should therefore probably also be followed. Moreover, the efficacy of fronto-temporo-parietal tDCS (20 min of 2 mA tDCS or sham stimulation once a day for 10 consecutive weekdays) was tested in the treatment of cognitive symptomatology in the early stages of psychosis [38].

The timing of the stimulation application is an important parameter that may condition the efficacy of the tDCS. In the frame of clinical trials, it would be determined based on electro-clinical variables. In the laboratory, for example with the acute ketamine model of psychosis transition, it would be essential to know a threshold value of the ongoing brain or large-scale network oscillopathies, from which a closed-loop device could trigger, uniquely or repetitively, the stimulator. The threshold value could be, for instance, the amplitude, the power, and/or the pattern of the aberrant oscillatory activities. Thereby, closed-loop neurostimulation would provide therapeutic stimulation only when necessary, a promising way for oscillotherapeutics [83] in a frame of personalized medicine. Early studies have shown that diverse conditions such as Parkinson’s disease [84], chronic pain [85], and intractable temporal lobe epilepsy [86] can benefit from a therapeutic closed-loop stimulation.
